# Do resting brain dynamics predict oddball evoked-potential?

**DOI:** 10.1186/1471-2202-12-121

**Published:** 2011-11-24

**Authors:** Tien-Wen Lee, Younger W-Y Yu, Hung-Chi Wu, Tai-Jui Chen

**Affiliations:** 1 Laureate Institute for Brain Research, Tulsa, Oklahoma, USA; 2 College of Medicine, Chang Gung University, Taoyuan County, Taiwan; 3 Yu's Psychiatric Clinic, Kaohsiung, Taiwan; 4 Kai-Suan Psychiatric Hospital, Kaohsiung, Taiwan; 5 Department of Psychiatry, E-DA Hospital, Kaohsiung County, Taiwan; 6 Department of Occupational Therapy, I-Shou University, Kaohsiung County, Taiwan

## Abstract

**Background:**

The oddball paradigm is widely applied to the investigation of cognitive function in neuroscience and in neuropsychiatry. Whether cortical oscillation in the resting state can predict the elicited oddball event-related potential (ERP) is still not clear. This study explored the relationship between resting electroencephalography (EEG) and oddball ERPs. The regional powers of 18 electrodes across delta, theta, alpha and beta frequencies were correlated with the amplitude and latency of N1, P2, N2 and P3 components of oddball ERPs. A multivariate analysis based on partial least squares (PLS) was applied to further examine the spatial pattern revealed by multiple correlations.

**Results:**

Higher synchronization in the resting state, especially at the alpha spectrum, is associated with higher neural responsiveness and faster neural propagation, as indicated by the higher amplitude change of N1/N2 and shorter latency of P2. None of the resting quantitative EEG indices predict P3 latency and amplitude. The PLS analysis confirms that the resting cortical dynamics which explains N1/N2 amplitude and P2 latency does not show regional specificity, indicating a global property of the brain.

**Conclusions:**

This study differs from previous approaches by relating dynamics in the resting state to neural responsiveness in the activation state. Our analyses suggest that the neural characteristics carried by resting brain dynamics modulate the earlier/automatic stage of target detection.

## Background

The oddball task is a broadly applied paradigm in the investigation of cognitive function. Target stimuli are presented amongst more frequent standard background stimuli. A distracter stimulus may also be used to ensure that the response is to the target rather than to the change from a background pattern. The waveform of elicited evoked-potential is thought to reflect central electrical activity related to a number of cognitive processes, such as attention allocation, stimulus evaluation and memory-based comparison, and has been studied in a variety of neurological and psychiatric disorders [1-9]. Oddball design is welcome because of its conceptual clarity, replicability, stability and inter-laboratory consistency [10-12]. The associated event-related potential (ERP) is composed of several components such as N1, P2, N2 and P3(00), indicating early/pre-attentive to late selective attention and cognitive processing. N1 and N2 are the post-stimulus negative deflections respectively peaking at around 100 ms and 200-300 ms, while P2 and P3 are the post-stimulus positive deflections respectively peaking at around 150-200 ms and 300 ms. Despite broad application, the determinants and moderators of each oddball ERPs component are still not fully understood.

The early (N1 and P2) components of oddball ERPs have been regarded to indicate automatic stimulus processing. They are influenced by early aspects of attention and orientation [13,14]. The N2 component may reflect the attended mismatch detection [15], whereas the P3 component is assumed to be an indicator of controlled processing [16]. Further, N2-P3 ERP complex has been reported to differentiate the infrequent targets and the frequent non-targets across different physical attributes of experimental stimuli [17-20]. A variety of psychological and biological factors may modulate the latency and amplitude of oddball ERPs. The psychological modulators comprise arousal, cognitive effort, task difficulty, experimental design, perceived emotion, meditation practice and even personality [21-25]. Many pharmacological agents, including ethanol, and genetic factors may also influence the profile of oddball ERPs [23,26-32]. Each component of oddball ERPs may correspond to a distinct psychological sub-process and may possess dissociable paths to interactive psycho-biological variables.

Among the modulators of oddball ERPs, the perspective of neural-neural interaction has been less addressed in the previous literature. Nevertheless, neural-neural interaction is critical in the theoretical framework to explain the generation of ERP. For example, previous studies have proposed that phase-resetting and phase synchronization between different frequency bands play a substantial role in the construction of early ERP [33,34]. Barry et al. found that the stimuli onset relative to the phase of brain dynamics may modulate the amplitude of oddball N1, P2 and P3 [35]. Regarding neural-neural interaction, whether the quantitative EEG (qEEG) indices can predict the oddball ERPs manifest over the vertex is still not clear. QEEG, in contrast to clinical EEG, is digital EEG information quantified using various precise and objective ways by computerized analytic and statistical techniques. However, twin studies have demonstrated moderate to high heritability in P300 and resting EEG power spectrum, and significant genetic correlation between P300 and background EEG power [36,37]. The observed common genetic influences on P300 amplitude and resting EEG support above conjecture that across-subject variance in resting neural dynamics may explain the variability of P300. Neural synchronization (e.g. regional power) in the resting state has been reported to correlate with performance on several neuro-psychological tasks, and even neuropsychiatric conditions [38-43]. Previous studies have proposed that the neuromatrix in the resting state may predict performance on attention tasks, as well as working memory tasks [44-46]. The agreement between structural (diffusion tensor imaging) and functional connectivity at a resting state has also been examined by cross-modal validation [47-49]. Furthermore, an early report by Romani et al. showed that pre-stimulus slow-wave activity differentiated the N2 latency and P3 amplitude elicited by the oddball paradigm, which supported the potential linkage of neural dynamics between the resting state and the activation state of target detection [50]. This study plans to investigate the relationship between cortical dynamics in resting state and the averaged oddball ERPs (as a surrogate of neural responsivity).

This study extends the previous understanding by incorporating resting brain dynamics into the study of auditory oddball ERPs. The resting neural informatics are actually irrelevant to the occurrence of oddball events, very different from previous approaches addressing brain dynamics at or after the occurrence of stimulus [33-35]. We are particularly interested in the qEEG indices of regional power at the fronto-parietal pathway associated with selective attention and working memory [44-46,51]. To the best of our knowledge, whether brain oscillation in the resting state can predict the oddball ERPs profile has never been assessed before. Since both gender and age may influence P300 topography and neural interaction in the resting state, this preliminary study restricts research sample to young females. The restricted sample may eliminate the potential interaction of gender and the differential impact of age on cortico-electrical activities and oddball ERPs [4,52-57].

## Methods

### Subjects

A total of 233 right-handed healthy young females, aged 19-22, were enrolled. All the participants were evaluated by licensed psychiatrists following a semi-structured interview process. The neurological and physical examinations were performed by licensed medical doctors. Those who had a history of substance abuse, psychiatric disease, or major medical or neurological disorder were excluded. Only those who had been medication-free (including birth control pills) for at least two weeks were enlisted. 218 participants were included in the final analysis. This project was approved by the local ethics committee conforming with Helsinki declaration. Informed consent was obtained from all participants prior to the commencement of the investigation.

### EEG recordings and analyses

All participants received a 3-minute conventional, eyes-closed, awake, digital EEG after a 5-minute habituation to the experimental environment (Brain Atlas III computer, Biologic System Company, Chicago). Recordings were in accord with the international 10-20 system, with linked ear reference, a 128 Hz sampling rate and impedance below 3 kΩ [58]. The artifact of vertical eyeball movement was detected from electrodes placed above and below the right eye, with the horizontal analog derived from electrodes placed at the left outer canthus. The common average reference method was adopted. We We defined four frequency bands as follows: delta 1 to 4 Hz, theta 4 to 8 Hz, alpha 8 to 12 Hz and beta 12 to 24 Hz. EEG segments with artifacts were deleted by an experienced EEG researcher.

An auditory oddball paradigm was used to elicit the oddball event-related potential (ERP). Fifty target tones (2,000 Hz, 80 dB SPL, probability 15.4%) and 275 non-target tones (1,000 Hz, 80 dB SPL) were interspersed in random order and presented binaurally. The tone pips were delivered at a stimulation rate of 1.3 tones/sec, with a 50-ms duration for each (10 ms rise/fall times). Subjects were required to count target stimuli while ignoring the frequent low-pitched tones. The data was discarded if the reported target number deviated more than 5 from the correct answer. The inter-trial interval of the auditory stimuli was varied in a 1,100-1,500 ms range (1,300 ms in average) to avoid expectancy effect. Other oddball ERPs detail can be referred to elsewhere [59].

As to the resting EEG signals, Fast Fourier Transform (FFT) was applied to consecutively non-overlapped and artifact-free segments of 20 sec to derive the mean EEG power for each electrode at a specified frequency band [60] (unit: μV^2^). Eighteen electrodes, including F7, F3, Fz, F4, F8, T3, C3, Cz, C4, T4, T5, P3, Pz, P4, T6, O1, Oz and O2, were included for further analysis with oddball ERPs.

### Statistical analyses

This study aims at investigating whether the quantitative EEG indices in the resting state predict oddball ERPs parameters. For the oddball waveforms at Fz, Cz and Pz, the amplitude and latency of N1, P2, N2 and P3 were quantified. Our preliminary analyses revealed a highly correlated pattern across Fz-oddball, Cz-oddball and Pz-oddball. We thus performed principal component analysis (PCA) to reduce the three oddball ERPs to a representative one, PCA-oddball. The amplitude and latency of N1, P2, N2 and P3 from the extracted PCA-oddball were then correlated (Pearson's correlation) with resting state spectral powers. For each test set in this study, the criterion for significance is thresholded at *P *< 0.05, two-tailed. We assume independence of each frequency band and perform the Bonferroni correction based on *P *= 1 - (1 - 0.05)^1/n^, where n equals the number of comparisons. The statistical threshold of the Bonferroni correction is 0.0028 (n = 18). Given that the electro-cortical activities are not completely independent, we report both *P *< 0.01 and *P *< 0.0028 in case the multiple comparison correction is too stringent.

For the correlation maps of resting dynamics and PCA-oddball showing significance, we used a multivariate method of partial least squares (PLS) to examine the spatial pattern of resting neuro-dynamics that predicted PCA-oddball. The PLS method was developed by McIntosh et al. and has been successfully applied in various neuroimaging studies, including EEG [61-63]. PLS is different from the voxel-wise approach in neuroimaging field that used to adopt multiple univariate statistical analyses. On the contrary, PLS is a multi-variate method to extract the distributed patterns of brain response that are related to task demands (categorical variables, Task PLS) or task performance (continuous variables, Behavioral PLS). In brief, PLS resorts to singular value decomposition (SVD) to compute the relation between 2 (or more) multivariate factors (e.g. from variance-covariance matrix) and yields a new set of covariance images that correspond to the strongest effects in the data. PLS is conceptually similar to canonical correlation analysis but optimizes covariance, not correlation. The outputs of PLS serve as the bases to weight the original multivariate factors to achieve optimization of the SVD factorization. The statistical assessment of PLS relies on non-parametric methods to provide the information of statistical strength and reliability of regional contribution by permutation test and bootstrap resampling, respectively. The variance-covariance matrix, in this study, is derived from oddball ERPs (218 subjects and 4 amplitudes or 4 latencies) and resting EEG (218 subjects and 18 electrodes). We utilized the Behavior PLS module, in contrast to Task PLS counterpart, to assess the saliencies of "behavior" (PCA-oddball) and "brain" (resting qEEG) [62]. The test of significance of PLS analysis is accomplished by permutation and bootstrapping for 2,500 and 500 times, respectively. The ratio of the salience to the bootstrap standard error is approximately equivalent to a z-score [64]. The spatial patterns with the squared singular value less than 10 percent of the total squared sum of all singular values were disregarded.

## Results

**Figure 1:**
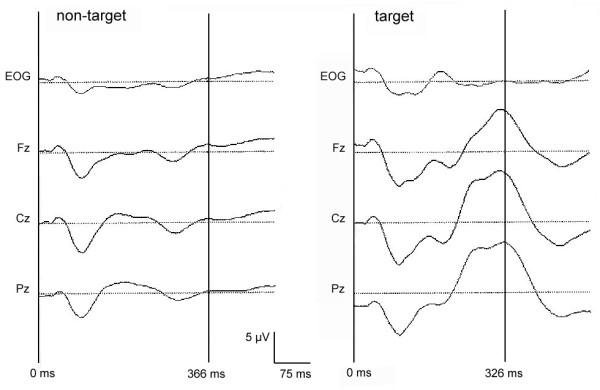
**The grand mean waveforms of oddball ERPs at Fz, Cz, Pz and EOG**. Time 0 indicates the stimulus onset.

**Figure 2:**
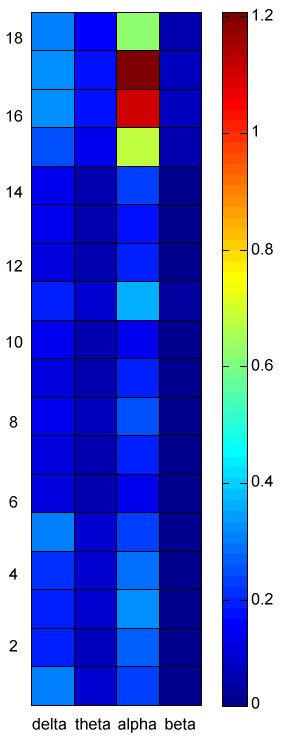
**The average mean powers of 18 electrode sites at delta, theta, alpha and beta frequencies of resting EEG**. The values are represented by color, with a color-bar at the right side. Ordinate is the EEG electrodes: 1. F7, 2. F3, 3. Fz, 4. F4, 5. F8, 6. T3, 7. C3, 8. Cz, 9. C4, 10. T4, 11. T5, 12. P3, 13. Pz, 14. P4, 15. T6, 16. O1, 17. Oz, 18. O2.

The grand mean waveform of oddball ERPs and resting EEG spectrum are illustrated in Figure 1 and 2, respectively. In Figure 1, the negative value of grand mean P2 is due to the relatively large variance in N2 and P2 latencies and hence, the individual P2 is masked by negative deflections in N1-P2 and P2-N2 segments. For the PCA-oddball, the percentage of the first eigenvalue relative to the summation of all eigenvalues ranges from 0.80 to 0.95 (mean 0.90). The detail of PCA-oddball and the N1, P2, N2, and P3 components of oddball ERPs are summarized in Table 1. The amplitude and latency of PCA-oddball are correlated with regional power derived from resting EEG. In the text henceforth, the prefix PCA- before oddball components will be discarded without loss of clarity. We notice aggregated patterns in which the regional power of the resting EEG is significantly correlated with N1 amplitude, N2 amplitude and P2 latency. For N1 latency, N2 latency, P2 amplitude and P3 latency/amplitude, the results are generally negative. The details are described below.

**Table 1:** The event-related components of the oddball task

	Frontal	Central	Parietal	Var (1st Eig)
N1	-3.42 (1.64)	-3.86 (1.71)	-3.15 (1.50)	0.94

P2	1.64 (1.37)	2.74 (1.70)	2.89 (1.64)	0.80

N2	-3.67 (2.38)	-3.96 (2.57)	-2.90 (2.09)	0.95

P3	6.77 (3.12)	8.33 (3.83)	8.38 (3.67)	0.89

				

N1	95.04 (13.96)	94.76 (13.32)	93.30 (14.57)	0.92

P2	179.65 (35.59)	178.61 (32.39)	178.30 (31.73)	0.89

N2	211.77 (32.48)	207.83 (32.34)	207.72 (35.11)	0.95

P3	324.39 (29.46)	320.59 (30.30)	321.53 (30.57)	0.87

Upper part: amplitude (μV), lower part: latency (ms), expressed as Mean (STD)

Var (1st Eig): variance explained by first principal component

N1 and N2 amplitudes are negatively correlated with regional power at the alpha band. Since we preserve the signs of negative peaks, our finding implies that the stronger the regional power at alpha in resting state, the greater the magnitude of the negative peaks at N1 and N2. The electrodes with significant correlations cover widespread brain regions, including frontal, temporal and parietal areas. The results of N1 and N2 amplitudes with regional power are summarized in Table 2.

**Table 2:** Correlation coefficients of oddball ERP and resting regional power across 18 channels at 4 frequency bands

	N1 Amplitude				N2 Amplitude				P2 Latency			
	**delta**	**theta**	**alpha**	**beta**	**delta**	**theta**	**alpha**	**beta**	**delta**	**theta**	**alpha**	**beta**

F7	-0.049	-0.124	-0.203*	-0.122	-0.127	-0.121	**-0.180**	-0.116	-0.110	-0.124	**-0.184**	-0.099

F3	-0.104	**-0.186**	**-0.176**	-0.132	-0.110	-0.109	-0.162	-0.127	-0.139	-0.173	**-0.183**	-0.112

Fz	-0.100	**-0.177**	-0.170	-0.123	-0.142	-0.137	-0.166	-0.108	-0.156	-0.150	-0.173	-0.084

F4	-0.088	-0.173	**-0.183**	-0.155	-0.083	-0.100	**-0.176**	-0.115	-0.109	-0.159	**-0.181**	-0.107

F8	-0.092	-0.156	**-0.197**	-0.147	-0.088	-0.097	**-0.176**	-0.130	-0.057	-0.133	**-0.174**	-0.116

T3	-0.051	-0.162	-0.206*	-0.111	-0.088	-0.127	-0.161	-0.170	-0.121	-0.149	-0.205*	-0.074

C3	-0.086	-0.155	-0.165	-0.134	-0.106	-0.093	-0.139	-0.143	-0.164	-0.173	-0.212*	-0.124

Cz	-0.077	-0.128	-0.135	-0.126	-0.150	-0.151	**-0.177**	-0.131	-0.187	-0.179	**-0.193**	-0.111

C4	0.007	-0.081	-0.155	-0.145	-0.048	-0.089	**-0.181**	-0.157	-0.124	-0.164	-0.220*	-0.143

T4	-0.050	-0.146	**-0.195**	-0.116	-0.022	-0.075	-0.207*	-0.146	-0.060	-0.121	-0.214*	-0.038

T5	-0.088	-0.139	-0.129	-0.144	-0.077	-0.111	-0.156	-0.122	-0.150	-0.160	**-0.186**	-0.122

P3	-0.089	-0.136	-0.213*	-0.172	-0.008	-0.066	-0.146	-0.158	-0.142	-0.140	-0.213*	-0.150

Pz	-0.116	-0.137	-0.167	-0.135	-0.089	-0.093	**-0.197**	-0.168	-0.173	-0.146	-0.254*	-0.173

P4	-0.056	-0.133	**-0.178**	-0.157	-0.028	-0.076	**-0.195**	-0.152	-0.152	-0.171	**-0.197**	-0.121

T6	-0.084	-0.161	-0.103	-0.105	-0.133	-0.137	**-0.191**	-0.128	-0.164	-0.142	-0.150	-0.094

O1	-0.108	-0.157	-0.141	-0.140	-0.099	-0.058	-0.117	-0.112	-0.189	-0.167	**-0.197**	-0.131

Oz	-0.075	-0.199	-0.164	-0.140	-0.141	-0.147	-0.161	-0.126	-0.169	-0.158	-0.160	-0.091

O2	0.012	-0.029	-0.151	-0.035	0.009	-0.019	-0.149	-0.092	-0.113	-0.149	**-0.198**	-0.117

*P *< 0.01 are printed in bold

*P < 0.0028

The latency of P2 is negatively correlated with the regional power at resting alpha band. This finding implies that stronger regional power at resting alpha would shorten the P2 latency. As in the analyses of N1/N2 amplitudes, most of the electrodes reveal significant correlations, indicating that the relationship is substantiated by a "global" neural characteristic, instead of regional specificity. The statistical threshold of the Bonferroni correction is the same as before, 0.0028. The results of the correlation of regional powers and P2 latency are displayed in Table 2. As to the details of the N1, P2, N2, P3 results not present in this report, interested readers may refer to the supplementary material (Tables S1 to S2, at http://www.websdj.idv.tw/kiki/rEEG_P300.pdf). The supplementary material also contains the results of multiple regression analysis with oddball ERPs and oscillatory powers as dependent and explanatory variables, respectively (Table S3).

Since the correlation maps at alpha frequency reveal a distributed, in opposition to focal, relation with N1/N2 amplitudes and P2 latency, associated spatial patterns are investigated using PLS. The PLS analyses comprise 2 categories of calculation, namely resting alpha-oddball amplitude and resting alpha-oddball latency. We notice that for each of the 2 PLS computations, only 1 prominent singular value satisfies the 10 percent criterion, with respective contribution 95.0 percent and 91.7 percent for oddball amplitude and latency. The permutation tests show that the respective probabilities to have singular values greater than the largest singular value are 0 and 0.0016. Concordant with the correlation maps, the saliencies associated with N1/N2 amplitudes as well as P2 latency are equipped with robust statistics, summarized in Table 3. It is interesting that for the two spatial patterns (from amplitudes and latencies of oddball ERPs) no regional specificity is noticed for all the electrodes at alpha frequency, summarized in Table 4. The contents of Table 3 and 4 in fact can be viewed as the weighting factors for oddball ERPs and electrodes that contribute to the optimized variance-covariance structures after SVD, correspondent to the largest singular values for the relationship of oddball amplitude - resting alpha and oddball latency - resting alpha. Together, the alpha oscillations that modulate N1/N2 amplitude and P2 latency reflect general characteristics of the brain, not confined to fronto-parietal network.

**Table 3:** The analyses of partial least squares on oddball ERP for resting EEG powers at alpha band

	Amplitude				Latency			
	**N1**	**P2**	**N2**	**P3**	**N1**	**P2**	**N2**	**P3**

Saliency	**0.665**	-0.189	**0.667**	-0.276	0.325	**0.902**	0.271	-0.087

*P *value	**0.001**	0.820	**0.001**	0.931	0.039	**10^-5 ^**	0.061	0.720

*P *< 0.01 are printed in bold

**Table 4:** The analytic results of partial least squares regarding the saliencies of electrodes at alpha frequency band for amplitudes and latencies of oddball ERP

	Amplitude	*P value*	Latency	*P value*
F7	-3.494	0.0002	-3.354	0.0004

F3	-3.276	0.0005	-3.507	0.0002

Fz	-3.278	0.0005	-3.317	0.0005

F4	-3.184	0.0007	-3.483	0.0002

F8	-3.309	0.0005	-3.352	0.0004

T3	-3.565	0.0002	-3.697	0.0001

C3	-3.047	0.0012	-3.881	0.0001

Cz	-3.195	0.0007	-3.925	< 10^-4^

C4	-3.607	0.0002	-4.391	< 10^-4^

T4	-3.705	0.0001	-4.249	< 10^-4^

T5	-3.305	0.0005	-4.671	< 10^-4^

P3	-3.857	0.0001	-3.942	< 10^-4^

Pz	-3.707	0.0001	-4.504	< 10^-4^

P4	-3.804	0.0001	-3.927	< 10^-4^

T6	-2.893	0.0019	-2.129	0.0166

O1	-1.646	0.0499	-3.334	0.0004

Oz	-3.131	0.0009	-2.708	0.0034

O2	-2.353	0.0093	-3.940	< 10^-4^

## Discussion

The oddball paradigm is a cognitive surrogate in probing the capability of target detection, which demands attention allocation, stimulus evaluation and comparison, and has been widely applied in a variety of neuro-psychiatric conditions. This study examines the hypothesis of whether the brain oscillation in the resting state, i.e. irrelevant to the active experiment, could predict the elicited oddball ERPs. Our design is different from that of event-related synchronization/desynchronization, which follows a triggering stimulus. Our analyses show that alpha power in the resting state is negatively correlated with the negativity of N1/N2 and the latency of P2. Without exception, the correlating trend (positive or negative) is consistent for all the significant qEEG indices in terms of each oddball ERPs component. The demonstrated aggregated correlation patterns indicate that the interaction between the resting versus activation conditions possesses spectral specificity. The finding of resting alpha oscillation and N1/N2/P2 is concordant with previous literature addressing the contribution of alpha dynamics to the generation of ERPs [33,34]. We do not notice any significant correlation between resting qEEG and P3 latency/amplitude, though. Contradictory to our prediction, our multivariate analysis of PLS reveals no topographical preference, such as fronto-parietal network, for the neural-neural interactions. Instead, it seems a global property of the brain reflected in resting neuro-dynamics that predicts the earlier component of P300.

N1 and P2 have been associated with lower-level perceptual processing and mental speed, while N2 is relevant to short-term memory and influenced by stimulus novelty [65,66]. Our analyses show that the stronger the alpha power in the resting and relaxed state, the greater the magnitude of the negative peaks at N1 and N2 of oddball ERPs. Although alpha power has been associated with an arousal level that may influence N1/N2 [21,67], arousal may not be an appropriate interpretation in this study since the oddball ERPs were acquired in a widely awake status and was independent of the eye-closed resting condition. An alternative and more straightforward account is that the value of alpha power during eyes-closed relaxation represents the degree of automatic neural orchestration/synchronization. It may be the property of innate synchronization, not necessarily via arousal, that modulates the amplitude of N1 and N2 in target detection. However, the possibility that the participants were at similar arousal level in both experimental conditions (i.e. resting and oddball) cannot be completely excluded.

Stronger regional power at alpha is observed to shorten the P2 latency. As with the widely distributed alpha oscillations in predicting N1/N2 amplitudes, the significant correlations spread over most of the electrodes, again indicating that the relation of resting and activation conditions is substantiated by a "global" neural characteristic. This observation is further endorsed by the PLS computation. The oddball P2 was reported to be correlated with response time [66,68,69], concordant with our observation at the latency dimension of P2. Although P2 was associated with mental speed, the scalp topography for P2 was similar in overt and covert responding conditions [70]. In addition, P2 was sensitive to task difficulty [25,68]. The psychological function of P2 may be pertinent to stimulus evaluation in the earlier processing stage rather than to the response production.

Even though all oddball ERPs components are relevant to the processing of the target, the components P2 and N2 have been regarded as different from P3. It was reported that the P2/N2 stage reflected automatic processing, whereas the P3 stage was capacity-limited, as suggested by the design manipulating task difficulty [71]. Coupled with our negative findings of P3, our results indicate that the neural characteristics carried by resting brain dynamics play a certain role in the earlier/automatic stage of target detection, with the stronger synchronization in resting relaxation, the higher or faster evoked potentials in the automatic processing stage. Our discovery is conceptually analogous to other reports showing that the neural property in the resting state has a fair predictability about various behavioral phenotypes, such as the performance on several neuro-psychological tasks, as well as the manifestation of neuropsychiatric diseases [38-46]. Recent studies have further suggested a correspondence between structural and functional connectivity at a resting state [47-49]. Together, the bridge between the resting alpha oscillation and the earlier components of oddball ERPs could be mediated by the hardwire organization of neural architecture.

Our finding at alpha frequency is intriguing since alpha oscillation has been related to a wide variety of cognitive processes. For example, alpha frequency was significantly correlated with the speed of information processing [72] and alpha power change has been observed in long-term memory, semantic processing and attention deployment [73-75]. Regional and long-range connectivity change at alpha frequency has also been noticed in executive functioning [76]. Synchronization of EEG alpha used to be regarded as an index of "idling" state since alpha power has been reported to be negatively correlated with brain activity, as reflected by metabolic and hemodynamic indices via positron emission tomography and functional magnetic resonance imaging, respectively [77-79]. However, recent reports observed increased alpha in creative thinking and/or in a state with higher internal processing demands [80,81]. Klimesch et al. proposed a theory of inhibition-timing that alpha synchronization is "functional" and reflects active, top-down, inhibitory control processes [82]. The spreading of cortical activation (such as the dynamics of topography of ERP) may be controlled by timely decreased inhibition, indicated by de-synchronization of alpha power, at the regions associated with particular mental processing. In other words, the titrated reduction of alpha control may shape the ERP complex. Our findings endorse and extend Klimesch et al.'s hypothesis in that the degree of innate (resting) alpha synchronization/de-synchronization may also influence cortico-electrical activity in activation state (such as oddball ERPs).

It has been suggested that ERP may be explained by superimposed oscillatory components, notably delta and theta components in oddball ERPs [83-85]. The proposition and observation have led to the theory of "oscillatory neural assemblies" to account for ERP generation [86]. Nevertheless, concordant with this study, pre-stimulus alpha activity has been noticed to modulate auditory oddball ERPs and visual ERPs [87,88]. Together with our findings and the inhibition-timing theory of alpha proposed by Klimesch et al. [82], there seem to be a cross-spectral interaction between the resting or pre-stimulus alpha and delta/theta components of the elicited ERP. Furthermore, the pre-stimulus spectral activity may serve as a mediator between the EEG activities in resting condition and in activation condition. It is thus worthwhile for future studies to design a unified method framework and brain model to delineate the relationship of cortico-electrical characteristics between different mental states and across different spectrum. Although our results highlight a global pattern at alpha rhythm, Bonferroni correction of multiple comparisons may be too stringent to mask regional contribution. Alternative statistical strategy such as False Discovery Rate can be considered in future studies to clarify the roles of specific brain regions in the generation of oddball ERPs.

It is noteworthy that our findings do not provide an exclusive account for the oddball ERPs. In fact, there are plenty of psychological and biological factors that modulate the oddball ERPs profiles. For example, it was suggested that N1 amplitude was accentuated in response to enhanced cortical arousal and increased top-down cognitive effort [21-23], and earlier component N1/P2 was affected by bi-aural competition [24]. Evidence suggested that N1 and P2 were different, with increased task difficulty imposing no effect on N1, but decreasing the amplitude of P2 [25]. N2/P3, the later component of oddball ERPs, was affected by several contextual factors in addition to attention engagement, such as perceived emotion, affective arousal, background music and even personality [17,89-91]. Both early and late oddball ERPs were influenced by practicing meditation [89]. The pharmacological perturbation of oddball ERPs is complicated. It was reported that captopril increased the N1-P2 component, vasopressin enhanced N2, and caffeine increased the amplitude of P3 [26-28], while ethanol affected both early and late ERP components of oddball ERPs [29,30]. Oddball ERPs was also differentiated by genetic factors, such as polymorphisms of serotonin transporter, nicotine receptor and apolipoprotein [23,31,32]. Whether the psychological, contextual, pharmacological and genetic modulators of oddball ERPs are mediated by the change in alpha oscillation is an important issue demanding further research to clarify.

Several studies have demonstrated that the baseline features immediately preceding stimulus onset can predict subsequent neural response or neuropsychological performance [92-94]. It should not be confused that the resting "baseline" in our design is not immediately preceding to the experimental event and has nothing to do with the trial-by-trial variation. Unlike the studies exploring N1/N2/P2 dipole sources of oddball ERPs, our positive findings are not restricted to fronto-parietal network or superior temporal plane [95-99]. Nevertheless, this study delineates a cortico-electrical linkage between brain informatics in resting and activation states. Our results complement the understanding of various modulators of oddball ERPs and imply that the resting alpha dynamics is one of the candidates that affect the earlier components (N1/P2/N2) [17,23,26-32,89-91]. Whether the resting dynamics also explain the earlier stages of ERPs of other experimental conditions is an issue of interesting theoretical implication.

## Conclusion

There are plenty biological and psychological modulators of oddball ERPs. The neuromatrix in the resting state has been reported to carry abundant information as an endo-phenotype. This study relates the brain dynamics in resting state to those in activation state of target detection. Our analyses suggest that the neural characteristics carried by resting brain modulate the earlier/automatic stage of target detection. The modulatory effects show spectral specificity at alpha band but no topographical preference.

## Competing interests

The authors declare that they have no competing interests.

## Authors' contributions

All the authors contributed to the conception and design of this project. Authors TJC and YWYY designed the study and refined the protocol. Author TWL managed the literature search and statistical analysis, and wrote the first draft of the manuscript. Authors TJC, YWYY and HCW executed the experiment protocol and undertook the EEG data collecting procedure, subject evaluation and quality control. All authors contributed to and have approved the final manuscript.
